# Deficiency of S100A8/A9 attenuates pulmonary microvascular leakage in septic mice

**DOI:** 10.1186/s12931-023-02594-0

**Published:** 2023-11-17

**Authors:** Jiang Yu, Boying Zhao, Qiangzhong Pi, Guoxiang Zhou, Zhe Cheng, Can Qu, Xiaowen Wang, Lingwen Kong, Suxin Luo, Dingyuan Du, Yongzheng Guo

**Affiliations:** 1 Division of Cardiology, The First Affiliated Hospital of Chongqing Medical University, Chongqing, 400016 China; 2 Department of Cardiothoracic Surgery, Chongqing Emergency Medical Center, Chongqing University Central Hospital, Chongqing University, Chongqing, 400010 China; 3 Chongqing Key Laboratory of Emergency Medicine, Chongqing, 400010 China; 4 Department of Respiratory Medicine, Southwest Hospital, Army Military Medical University, Chongqing, P.R. China; 5 Department of Cardiology, Chongqing University three Gorges Hospital, Chongqing, 404199 China; 6 Department of Pharmacy, The First Affiliated Hospital of Chongqing Medical University, Chongqing, 400016 China; 7 Department of Cardiothoracic Surgery, The First Affiliated Hospital of Chongqing Medical University, Chongqing, 400016 China

**Keywords:** S100A8/A9, sepsis, Pulmonary inflammation, Vascular leakage, Acute lung injury

## Abstract

**Background:**

We have reported a positive correlation between S100 calcium-binding protein (S100) A8/S100A9 and sepsis-induced lung damage before. However, limited knowledge exists concerning the biological role of S100A8/A9 in pulmonary vascular endothelial barrier dysfunction, as well as the diagnostic value of S100A8/A9 in sepsis.

**Methods:**

Sepsis was induced in C57BL/6J mice and S100A9-knockout (KO) mice through the cecal ligation and puncture (CLP). Pulmonary vascular leakage was determined by measuring extravasated Evans blue (EB). Reverse transcription polymerase chain reaction and the histological score were used to evaluate inflammation and lung injury, respectively. Recombinant S100A8/A9 (rhS100A8/A9) was used to identify the effects of S100A8/A9 on endothelial barrier dysfunction in human umbilical vein endothelial cells (HUVECs). Additionally, the diagnostic value of S100A8/A9 in sepsis was assessed using receiver operating characteristic.

**Results:**

S100A8/A9 expression was up-regulated in the lungs of CLP-operated mice. S100A9 KO significantly reversed CLP-induced hypothermia and hypotension, resulting in an improved survival rate. S100A9 KO also decreased the inflammatory response, EB leakage, and histological scores in the lungs of CLP-operated mice. Occludin and VE-cadherin expressions were decreased in the lungs of CLP-operated mice; However, S100A9 KO attenuated this decrease. Moreover, CLP-induced signal transducer and activator of transcription 3 (STAT3) and p38/extracellular signal-regulated kinase (ERK) signalling activation and apoptosis were mitigated by S100A9 KO in lungs. In addition, rhS100A8/A9 administration significantly decreased occludin and VE-cadherin expressions, increased the phosphorylated (p)-ERK/ERK, p-p38/p38, and B-cell leukaemia/lymphoma 2 protein (Bcl-2)-associated X protein/Bcl-2 ratios in HUVECs.

**Conclusion:**

The present study demonstrated S100A8/A9 aggravated sepsis-induced pulmonary inflammation, vascular permeability, and lung injury. This was achieved, at least partially, by activating the P38/STAT3/ERK signalling pathways. Moreover, S100A8/A9 showed the potential as a biomarker for sepsis diagnosis.

**Supplementary Information:**

The online version contains supplementary material available at 10.1186/s12931-023-02594-0.

## Introduction

Sepsis is now considered a severe disruption of the host innate immune system’s homeostasis caused by microbial pathogen infections. Its prevalence in intensive care units reaches a substantial 20.6% [1, 2]. Despite significant advances in the diagnosis and treatment of sepsis in recent years, the mortality rate remains high at 35.5% [2]. Additionally, sepsis imposes a significant burden on healthcare expenses [3, 4]. The lungs are particularly vulnerable during sepsis, with acute respiratory distress syndrome (ARDS) being a frequent and fatal consequence [5, 6]. Clinical manifestations of sepsis-induced ARDS include dyspnoea, progressive hypoxaemia, pulmonary oedema, and consequent noncardiogenic respiratory failure. These symptoms arise from an amplified inflammatory response, disruption of the alveolar-capillary barrier, and increased permeability of lung microvasculature [7–9]. Among these factors, pulmonary vascular leakage is considered a primary contributor to ARDS and mortality in sepsis. It results in excessive fluid leakage into the interstitium and alveoli, compromising the effective exchange of oxygen and carbon dioxide required for normal respiration [9]. Regrettably, there are currently no effective therapeutic approaches specifically targeting the improvement of pathological vascular leakage in sepsis, and the mechanisms underlying this leakage are yet to be elucidated.

S100 calcium-binding proteins A8 (S100A8) and A9 (S100A9), also known as myeloid-related protein (Mrp) 8 and Mrp14, are members of the calcium-binding S100 protein family. These proteins play a crucial role in inflammation and inflammation-associated tissue damage [10]. Under in vivo conditions, S100A8/A9 often form a stable heterodimer, which is essential for their intracellular function as homodimers lack stability [10]. Previous research has reported that S100A8/A9 primarily exert their effects by binding to toll-like receptor 4 (TLR4) or the receptor for advanced glycation end products [10, 11]. Currently, S100A8/A9 is identified as a valuable diagnostic and prognostic biomarker for inflammation-associated diseases [12]. Several studies, including from our lab, have revealed that S100A9 is up-regulated in lung tissue in mice models of sepsis, and targeting S100A9 function attenuates sepsis-induced lung damage [13]. However, there is limited knowledge regarding the specific role of S100A8/A9 in regulating pulmonary vascular integrity during sepsis.

The maintenance of endothelial barrier integrity relies on tight and adherens junctions molecules, such as occludin and vascular endothelial (VE)-cadherin, respectively [14]. Disruption of VE-cadherin is sufficient to increase the paracellular permeability, resulting in microvascular leakage and tissue oedema [15, 16]. Multiple studies have demonstrated the critical role of extracellular signal-regulated kinase (ERK)-1/2 and p38 mitogen-activated protein kinase (MAPK) signalling in regulating pulmonary vascular endothelial hyperpermeability during the development of acute lung injury [17–19]. A recent study discovered that S100A8/A9 could activate ERK-1/2 signalling by binding to TLR4 in the heart tissues of mice with sepsis [20]. Furthermore, our previous study revealed that S100A8/A9 increased p38 and p65 phosphorylation in the aorta of aged mice [21]. Based on these findings, it was speculated that S100A8/A9 play a crucial role in the disruption of endothelial barrier integrity during sepsis, and targeting the S100A8/A9 function might improve the prognosis of sepsis by improving vascular permeability.

Due to the embryonic lethality observed in S100A8-deficient mice and the lack of S100A8/A9 protein expression in S100A9-deficient mice [22], S100A9 knockout (KO) mice were used in this study to explore the role of S100A8/A9 in sepsis-induced pulmonary vascular hyperpermeability. The influence of S100A8/A9 on the inflammation to gain further insights into the underlying mechanisms of sepsis-induced lung injury was investigated. Moreover, the clinic diagnostic value of S100A8/A9 in sepsis was assessed using receiver operating characteristic.

## Materials and methods

### Animals

The C57BL/6J wild-type (WT) mice used in this study were procured from Hunan Slack Jingda Laboratory Animal Co., Ltd, China (usage certificate number: SCXK 2019-0004). The global S100A9 heterozygotes (S100A9^+/−^) mice were provided by Cyagen Biosciences Inc. (Suzhou, China). The S100A9 heterozygote mice were bred together to produce homozygotes S100A9 KO mice (S100A9^−/−^) (Supplemental Fig. 1). Genotyping of mice was performed using polymerase chain reaction (PCR) with tail deoxyribonucleic acid (DNA) according to the manufacturer’s instructions. The genotyping primers for S100A9 KO were as follows: forward (F) (5′-GTATATGTGGAGGGAAGCTGTCTC-3′) and reverse (R) (5′- GTGAAAGGAGGCAGAAAGGACATG-3′). The genotyping primers for S100A9 WT were as follows: F (5′-CAAAGTCCTAGTGCCCACGGC-3′) and R (5′-GTGAAAGGAGGCAGAAAGGACATG-3′). All mice were housed under controlled conditions with a temperature of 23.6℃, a relative humidity of 68.1%, a 12-h light/dark circle, and food and water ad libitum at the Experimental Animal Centre of Chongqing Medical University. All animal procedures were approved by the Animal Care and Use Committee of Chongqing Medical University and performed in accordance with the National Institutes of Health Guidelines for the Use of Laboratory Animals.

### Cecal ligation and puncture operation

Male WT and S100A9 KO mice, aged 8-10 weeks, were randomly selected for the experiments. The polymicrobial sepsis model was induced using the cecal ligation and puncture (CLP) technique as described previously [23–25]. The mice were anesthetised with a 1% pentobarbital sodium (0.05 mL/10g) solution. A 1-cm midline laparotomy was performed, and the abdomen was shaved. The cecum was exposed, and a tight ligation was applied to its middle portion using a 4-0 silk suture. The cecum was then perforated twice using a 21-gauge needle, and slight pressure was applied to extrude 1 mm of faecal matter from each puncture hole. Finally, the cecum was returned to the central abdominal cavity. The abdominal incision of the mice was closed, and 1 mL of sterile 0.9% saline was administered subcutaneously. The mice were placed on a thermostatic pad for 2 h to facilitate their body temperature recovery from anaesthesia. Sham controls underwent the same surgical procedures but without ligation or puncture. The rectal temperature of the mice was recorded every 2 h using a rectal thermometer throughout the observation period. Blood pressure in the carotid artery was measured using a catheter attached to a Multichannel Physiological Recorder (BIOPAC, USA) with a pressure sensor before the mice were sacrificed.

### Lung vascular leak assessment

Lung microvascular leakage was assessed by measuring the extravasated Evans blue (EB) dye, as described previously [26]. EB (0.5%, 0.01 mL/g) was injected into the mice via the tail vein 30 min before sacrificing them. Subsequently, the lungs were perfused with 100 mL of phosphate-buffered saline (PBS) through the right ventricle to remove the intravascular dye. Perfusion was continued until the fluid from the left atrium became colourless. The upper lobes of the left lung were collected, air-dried, and weighed. These samples were then incubated with 500 µL of formamide at 55 °C for 48 h to extract EB from the lung tissues. After centrifugation at 2000 g for 10 min, the absorbance of the supernatants was quantitated spectrophotometrically at 610 nm. A blank containing an equal volume of formamide was used for reference. The concentration of extravasated EB in the lung was calculated using a linear standard curve.

### Histopathology and lung injury score

The upper lobes of the left lung were collected 12 h after CLP or sham surgery. The collected lung tissues were fixed in 4% paraformaldehyde, embedded in paraffin, and cut into 4-µm thick sections. These sections were then stained using a haematoxylin-eosin staining kit as per the manufacturer’s instructions. The extent of histological lung injury was quantified using a scoring system [27]. In this system, each of the five independent variables was assigned a weight based on its relevance, as determined by the Committee. The sum of these weighted variables was used to generate a lung injury score, which was then normalised to the number of fields evaluated. The resulting injury score was multiplied by 10 to obtain a continuous value ranging from zero and 10 (inclusive).

### Quantitative reverse transcription PCR (RT-PCR)

Total ribonucleic acid (RNA) was extracted from lung tissue using the TRIzol total RNA extraction kit (Solarbio Biotechnology, China) according to the manufacturer’s protocol. Subsequently, complementary DNA (cDNA) was synthesised using the cDNA reverse kit (Cat #RT001, ESscience Biotech, China) as per the manufacturer’s instructions. RT-PCR was performed on a CFX96™ Real-Time system (Bio-Rad Laboratories, Inc., USA) using 2× Universal SYBR Green Fast qPCR Mix (Cat #RK21203, Abclonal, China). The gene expression levels were normalised to β-actin using the delta-delta Ct (2^ΔΔCT^) relative quantification method. The PCR primer sequences used for measuring gene expression are presented below (F, R): *β-actin mouse* (TGGAATCCTGTGGCATCCATGAAAC, TAAAACGCAGCTCAGTAACAGTCCG); *tumour necrosis factor* (*TNF*)*-α mouse* (CTTAGACTTTGCGGAGTCCG, ACAGTCCAGGTCACTGTCCC); *interleukin* (*IL*)*-1β mouse* (GACAACTGCACTACAGGCTCC, AGGCCACAGGTATTTTGTCG); *monocyte chemoattractant protein* (*MCP*)*-1 mouse* (CCACTCACCTGCTGCTACTCATTC, CTTCTTTGGGACACCTGCTGCTG).

### Immunofluorescence staining

The lung tissue sections were deparaffinised using xylene, followed by dehydration with an ethanol solution. Antigen retrieval was performed by boiling the sections in sodium citrate (pH 6.0) in a microwave oven. Immunofluorescence staining of lung tissue was performed as described previously [19]. The sections were washed with PBS and sealed with 10% goat serum. Subsequently, the sections were incubated overnight at 4℃ with VE-cadherin (Cat #ab33168, Abcam) or occludin (Cat #27260-1-AP, Proteintech Group, China) antibody diluted in PBS. After washing with PBS, the sections were incubated at room temperature for 1 h with fluorescein iso-thiocyanate/cyanine 3-conjugated fluorescent secondary antibodies and stained with 4′,6-diamidino-2-phenylindole. The Images were visualised under a fluorescence microscope (Leica Camera, Germany) and quantified using ImageJ software.

### Cell culture and treatment

Primary human umbilical vein endothelial cells (HUVECs) were cultured in endothelial cell medium (Cat#1001, Sciencell, CA, USA) and incubated in a 5% carbon dioxide humidified incubator. HUVECs were seeded in six-well plates and allowed to reach 90% confluence. Subsequently, the cells were treated with 2.0 µg/mL of human recombinant S100A8/A9 (Cat #HY-P71076, MedChemexpress, US) or PBS for 2 h, as reported previously [28].

### Western blotting

The right lung lobes weighing 20 mg from each group of mice were lysed with radioimmunoprecipitation assay lysis buffer supplemented with 1% protease and phosphatase inhibitors (Beyotime Biotechnology, China). After centrifugation at 12,000 rpm for 15 min at 4 °C, the protein supernatant was quantified, diluted with 5× loading buffer, and heated at 100 °C for 10 min. A total of 40 µg of protein per sample were separated using sodium dodecyl-sulphate polyacrylamide gel electrophoresis and transferred onto polyvinylidene difluoride membranes. Subsequently, the membranes were blocked with 5% skim milk for 1.5 h at room temperature and then incubated with the primary antibodies overnight at 4℃. After washing with Tris-buffered saline with 0.1% Tween® 20 detergent to remove the unbound antibodies, the membranes were incubated with corresponding secondary antibodies for 1.5 h at room temperature. Finally, the protein bands were visualised using an electrochemiluminescence imaging system (Bio-Rad Laboratories, Inc., USA). The intensities of protein bands were quantified using ImageJ software. The primary antibodies used in Western blotting included: β-actin (Cat # GB15001, Servicebio Biotechnology, China), S100A8/A9 (Cat #ab33168, Abcam), occludin (Cat #27260-1-AP, Proteintech Group, China), signal transducer and activator of transcription 3 (STAT3) (Cat # 12640S, CST), phosphorylated (p)-STAT3 (Cat #9145S, CST), p38 (Cat #14064-1-AP, Proteintech Group, China), p-p38 (Cat # AF4001, Affinity), ERK (Cat #9102S, CST), p-ERK (Cat #9101S, CST), B-cell leukaemia/lymphoma 2 protein (Bcl-2)-associated X protein (Bax) (Cat # 50599-2-Ig, Proteintech Group, China), Bcl-2 (Cat #BF9103, Affinity).

### Bioinformatics analysis

Two datasets, namely GDS4273, were obtained from the Gene Expression Omnibus database (http://www.ncbi.nlm.nih.gov/geo/) to evaluate the diagnostic value of S100A9 in patients with sepsis. The diagnostic value of S100A9 was analysed using the “pROC” R package and visualised using the “sggplot2” R package.

### Statistical analysis

All experimental data are presented as the mean ± standard error of the mean. Statistical analysis and generation of graphs were performed using GraphPad Prism 9.0 software. After the normality test and homogeneity test of variances, the Student’s t-test was used to analyse differences between two groups. For comparisons among four groups, two-way analysis of variance and Tukey’s test were employed. Survival differences were assessed using the Kaplan–Meier survival curve and analysed using the log-rank test. Statistical significance was set at P < 0.05.

## Results

### S100A9 KO protects against polymicrobial sepsis in mice

**Fig. 1:**
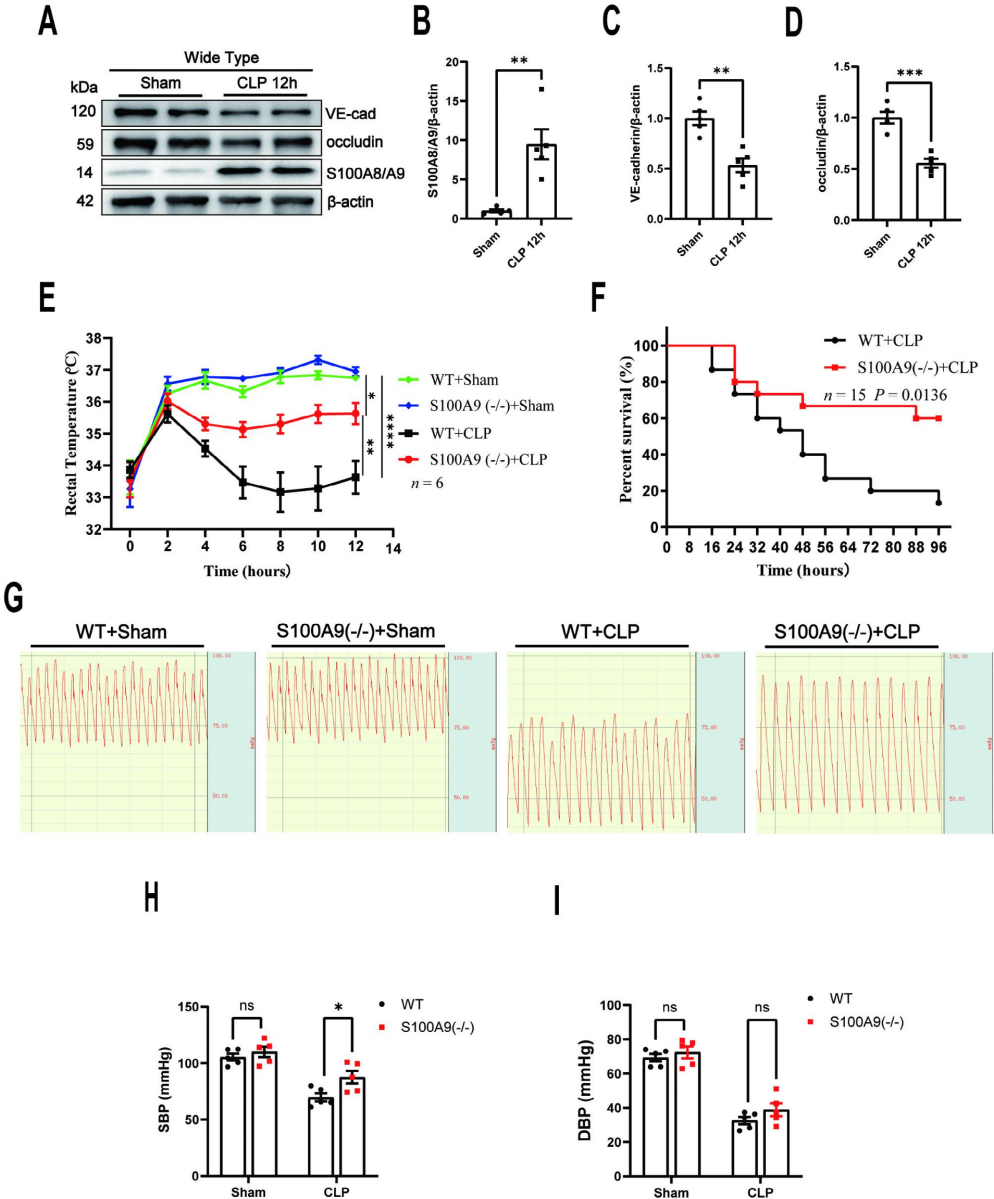
Elevated S100 calcium-binding protein (S100) A8/A9 levels are associated with pulmonary vascular hyperpermeability and disease severity in polymicrobial sepsis. **(A)** S100A8/A9, occludin, and VE-cadherin protein expression levels in the lungs of WT mice were assessed using Western blotting 12 h after CLP surgery. The statistical results of S100A8/A9, occludin, and VE-cadherin are illustrated in **(B)**, **(C)**, and **(D)** respectively (n = 5). **(E)** The body temperatures were assessed using a rectal thermometer 12 h after CLP/sham surgery (n = 6). **(F)** Survival rates among the WT and S100A9 KO mice after CLP surgery were compared using the Kaplan–Meier test (n = 15, three independent experiments). **(G)** Carotid pressures were measured 12 h after surgery and the related statistical results are presented in **(H-I)** (n = 5). S100, S100 calcium-binding protein; VE, vascular endothelial; WT, wide-type. KO, knockout. CLP, cecal ligation and puncture. VE-cad, VE-cadherin. The data are presented as the mean ± standard error of the mean. ns, no significant difference. *P < 0.05; **P < 0.01; ***P < 0.001; ****P < 0.0001

The S100A8/A9, occludin, and VE-cadherin protein expression levels in the lungs of CLP-induced mice with sepsis were measured to explore the functional role of S100A8/A9 in pulmonary vascular hyperpermeability in sepsis. The results demonstrated a significant increase in S100A8/A9 expression in the lungs after CLP at 12 h, whereas occludin and VE-cadherin exhibited a significant decrease compared with the sham group (n = 5) (Fig. 1A-D). S100A9 KO mice were constructed and subjected to CLP to further confirm the involvement of S100A8/A9 in the pathogenesis and development of sepsis. After CLP, S100A9 KO mice exhibited a reversal of hypothermia and hypotension, and a significantly improved survival rate compared with WT mice (n = 6 for Figs E and n = 5 for Figs F-I) (Fig. 1E-I).

### S100A9 KO attenuates CLP-induced pulmonary inflammation, vascular leakage, and acute lung injury

**Fig. 2:**
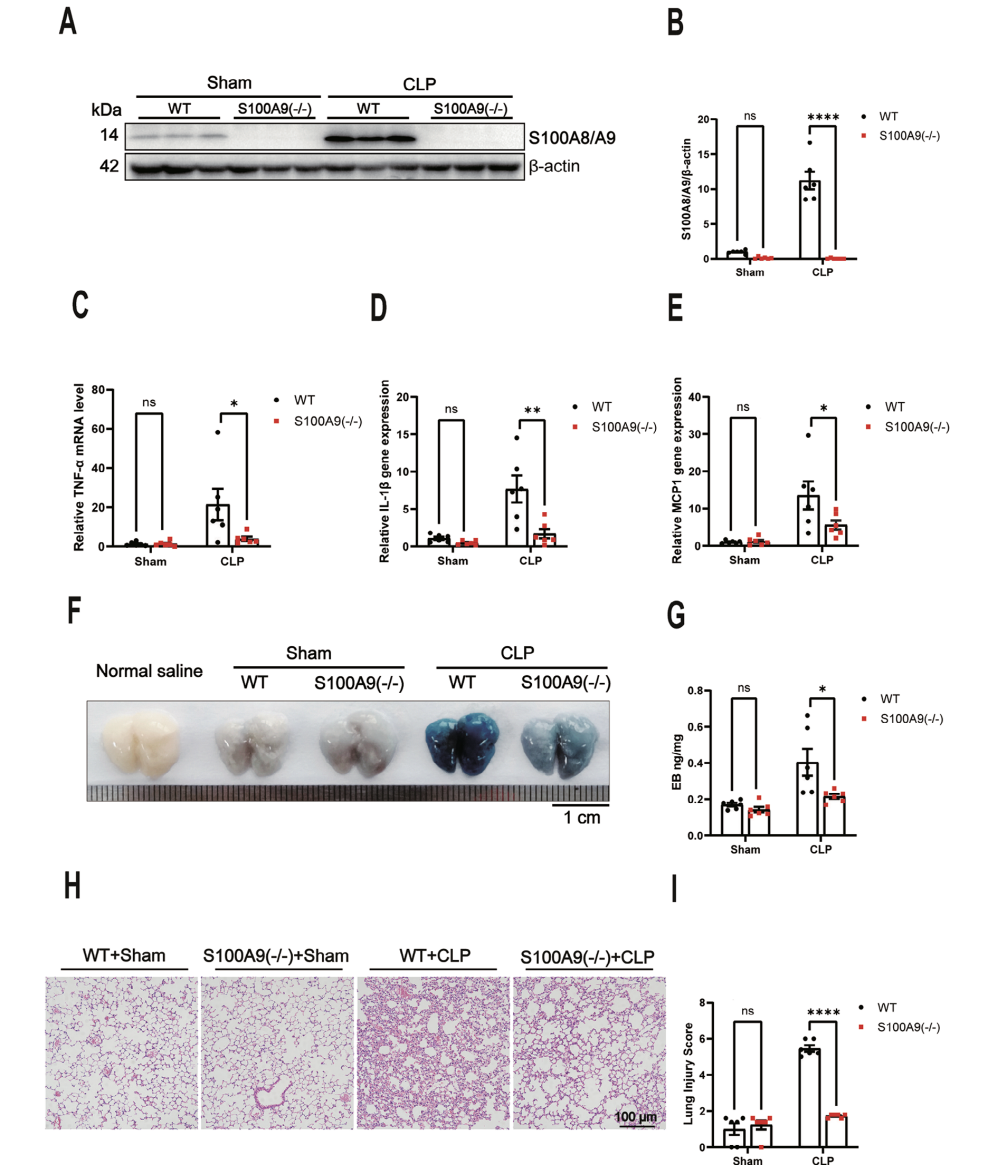
S100A9 knockout attenuates CLP-induced pulmonary inflammation, vascular leakage, and acute lung injury. **(A)** The S100A8/A9 protein expression in the lungs of mice was determined using Western blotting 12 h after CLP surgery (n = 6). The statistical results are presented in **(B)**. **(C-E)** The TNF-α **(C)**, IL-1β **(D)**, and MCP-1 **(E)** mRNA levels in the lungs of mice were analysed using RT-PCR 12 h after CLP surgery (n = 6). **(F)** The levels of EB inundated the lung tissues in mice after CLP surgery (n = 6). The statistical results of lung EB absorbance at 610 nm are presented in **(G)**. **(H)** The lung injury score was evaluated using a scoring system, as described in the Methods (n = 6). The statistical results of the histological score were exposed in **(I)**. S100, S100 calcium-binding protein; CLP, cecal ligation and puncture; TNF, tumour necrosis factor; IL, interleukin; MCP-1, monocyte chemoattractant protein; mRNA, messenger ribonucleic acid; RT-PCR, reverse transcription polymerase chain reaction; EB, Evans blue. The data are presented as the mean ± standard error of the mean. ns, no significant difference. *P < 0.05; **P < 0.01; ****P < 0.0001

The presence of S100A8/A9 protein in the lungs of S100A9 KO mice after CLP surgery was initially investigated to assess the significance of S100A8/A9 heterodimers, which are essential for their physiological function due to the instability of S100A8 or S100A9 homodimers. Consistent with our expectations, S100A8/A9 protein was not detected in the lungs of S100A9 KO mice (n = 6) (Fig. 2A-B). In consistently, RT-PCR analysis revealed a significant reduction in the messenger RNA levels of TNF-α, interleukin-1β, and MCP-1 in the lungs of S100A9 KO mice compared with WT mice after CLP surgery (n = 6) (Fig. 2C-E). In addition, our findings demonstrated a significant decrease in sepsis-induced pulmonary vascular leakage in S100A9 KO mice, as evidenced by the reduced EB extravasation in the lung (n = 6) (Fig. 2F-G). Furthermore, histological examination using H&E staining revealed a significantly lower lung injury score in S100A9 KO mice compared with WT mice 12 h after CLP surgery (n = 6) (Fig. 2H-I). Collectively, these results indicate that S100A8/A9 promotes the development of pulmonary vascular leakage and acute lung injury during sepsis.

### S100A9 KO attenuates CLP-induced decrease in occludin and VE-cadherin expression in the lungs

**Fig. 3:**
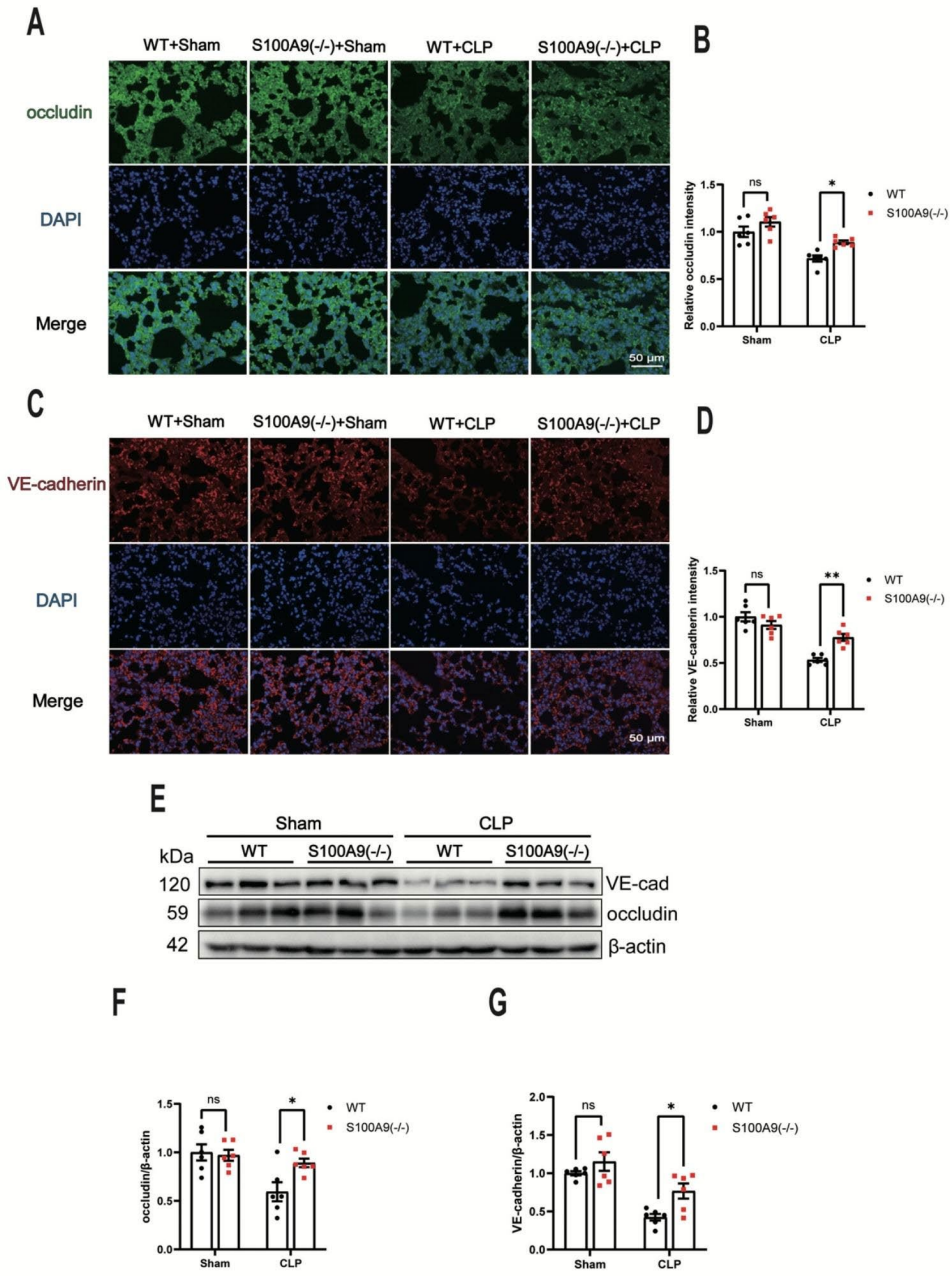
S100A9 knockout attenuates CLP-induced decrease in occludin and VE-cadherin levels in the lungs. **(A)** Representative immunofluorescent staining images of occludin (green) and DAPI (blue) in lung tissues of mice 12 h after CLP surgery (n = 6). The statistical results are presented in **(B)**. **(C)** Representative immunofluorescent staining images of VE-cadherin (red) and DAPI (blue) in lung tissues of mice 12 h after CLP surgery (n = 6). The statistical results are illustrated in **(D)**. **(E)** The occludin and VE-cadherin protein expressions in mice lungs were determined using Western blotting 12 h after CLP surgery (n = 6). The statistical results are presented in **(F)** and **(G)**. S100, S100 calcium-binding protein; CLP, cecal ligation and puncture; VE, vascular endothelial; DAPI, 4′,6-diamidino-2-phenylindole. Data are presented shown as the mean ± standard error of the mean. ns, no significant difference. *P < 0.05; **P < 0.01

Disruption of tight and adherens junctions molecules impairs endothelium barrier function. Therefore, the effects of S100A9 KO on occludin and VE-cadherin protein expression in the lungs following CLP surgery were explored. Immunofluorescent staining demonstrated a decrease in VE-cadherin and occludin expression in the lung vascular of WT mice 12 h after CLP surgery (n = 6) (Fig. 3A-D). In contrast, S100A9 KO significantly attenuated CLP sepsis-induced reduction of occludin and VE-cadherin levels in the lungs (n = 6) (Fig. 3A-D). Consistently, Western blotting also revealed that S100A9 KO significantly rescued the occludin and VE-cadherin expression in lungs of S100A9 KO mice (n = 6) (Fig. 3E-G). Those findings suggest that S100A9 KO mitigate pulmonary vascular leakage via prompting tight and adherent molecules expression.

### S100A9 KO suppresses CLP-induced p38/STAT3/ERK signalling activation and apoptosis in lungs

**Fig. 4:**
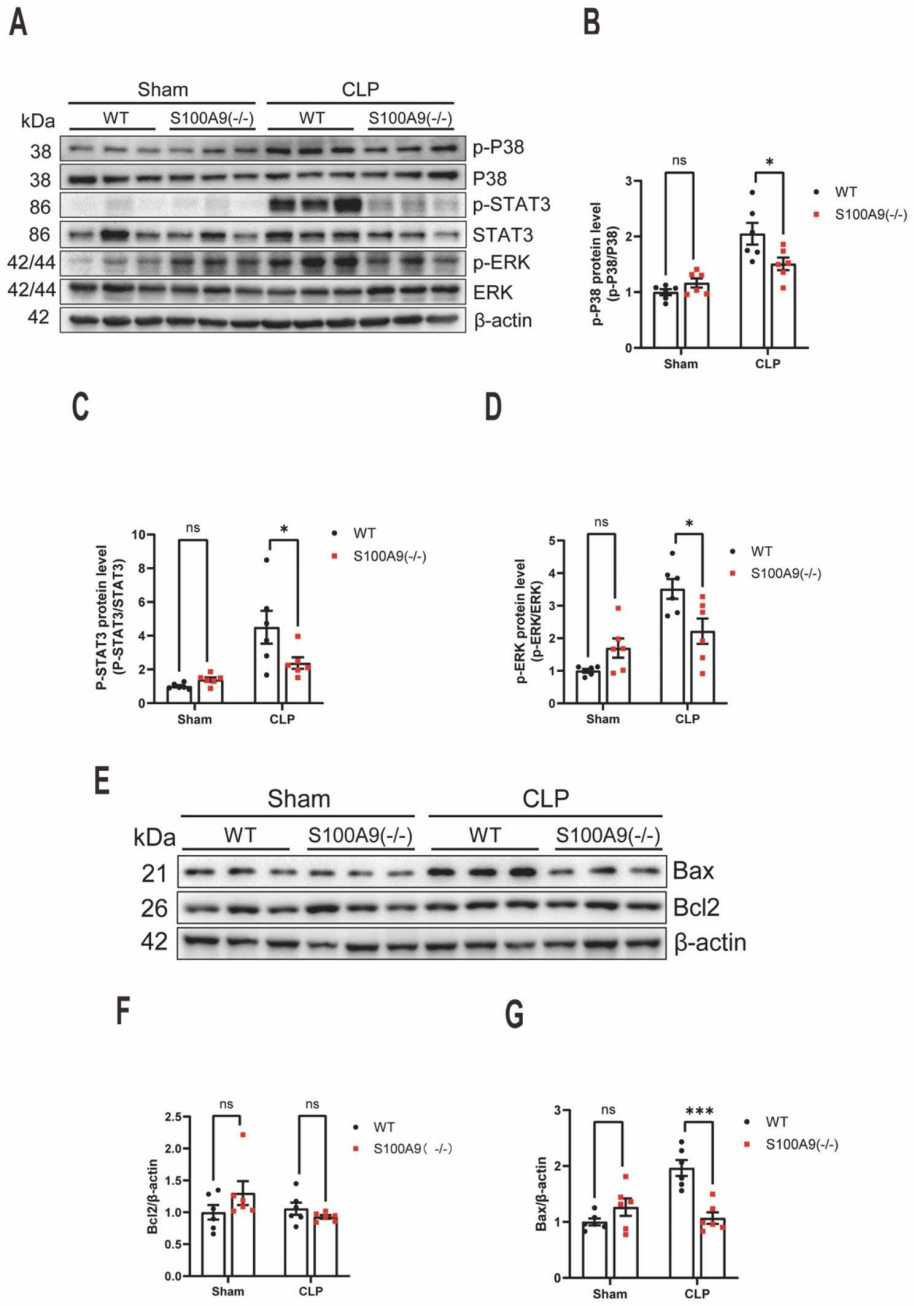
S100A9 knockout suppresses CLP-induced p38/STAT3/ERK signalling activation and apoptosis in the lungs. **(A)** Western blotting analysis of p38/STAT3//ERK signalling in the lung tissues of mice 12 h after CLP surgery (n = 6). The statistical results are presented in **(B-D)**. **(E)** Western blot analysis of Bax and Bcl-2 expressions in the lung tissues of mice 12 h after CLP surgery (n = 6). The statistical results are presented in **(F)**. S100, S100 calcium-binding protein; CLP, cecal ligation and puncture; STAT3, signal transducer and activator of transcription 3; ERK, extracellular signal-regulated kinase; Bax, B-cell leukaemia/lymphoma 2 protein (Bcl-2)-associated X protein. The data are presented as the mean ± standard error of the mean. *P < 0.05; ***P < 0.001

p38/STAT3/ERK signalling are interconnected and contribute to the complex regulation of septic inflammation [29]. Moreover, p38/ERK-mediated proteolytic disorganisation of occludin and VE-cadherin and endothelial cell apoptosis have also been implicated in the pathogenesis of sepsis-induced pulmonary vascular leakage [30, 31]. It was observed that S100A9 KO attenuated CLP-induced p38/STAT3/ERK signalling activation in lungs, as evidenced by decreased p38, STAT3 and ERK phosphorylation (n = 6) (Fig. 4A-D).

Furthermore, parenchyma and vascular endothelial cell apoptosis have been implicated in the pathogenesis of sepsis-induced acute lung injury [32]. Therefore, Bax and Bcl-2 protein levels in the lungs of CLP sepsis mice were examined (n = 6) (Fig. 4E-G). Although Bcl2 expression was not significantly affected by S100A9 KO, it was observed that S100A9 KO decreased the pro-apoptotic protein Bax expression(n = 6) (Fig. 4E-F). Together, those data showed that S100A8/A9 may function via p38/STAT3/ERK and apoptosis signalling in sepsis-induced pulmonary vascular leakage.

### S100A8/A9 promotes endothelial barrier dysfunction on HUVECs

**Fig. 5:**
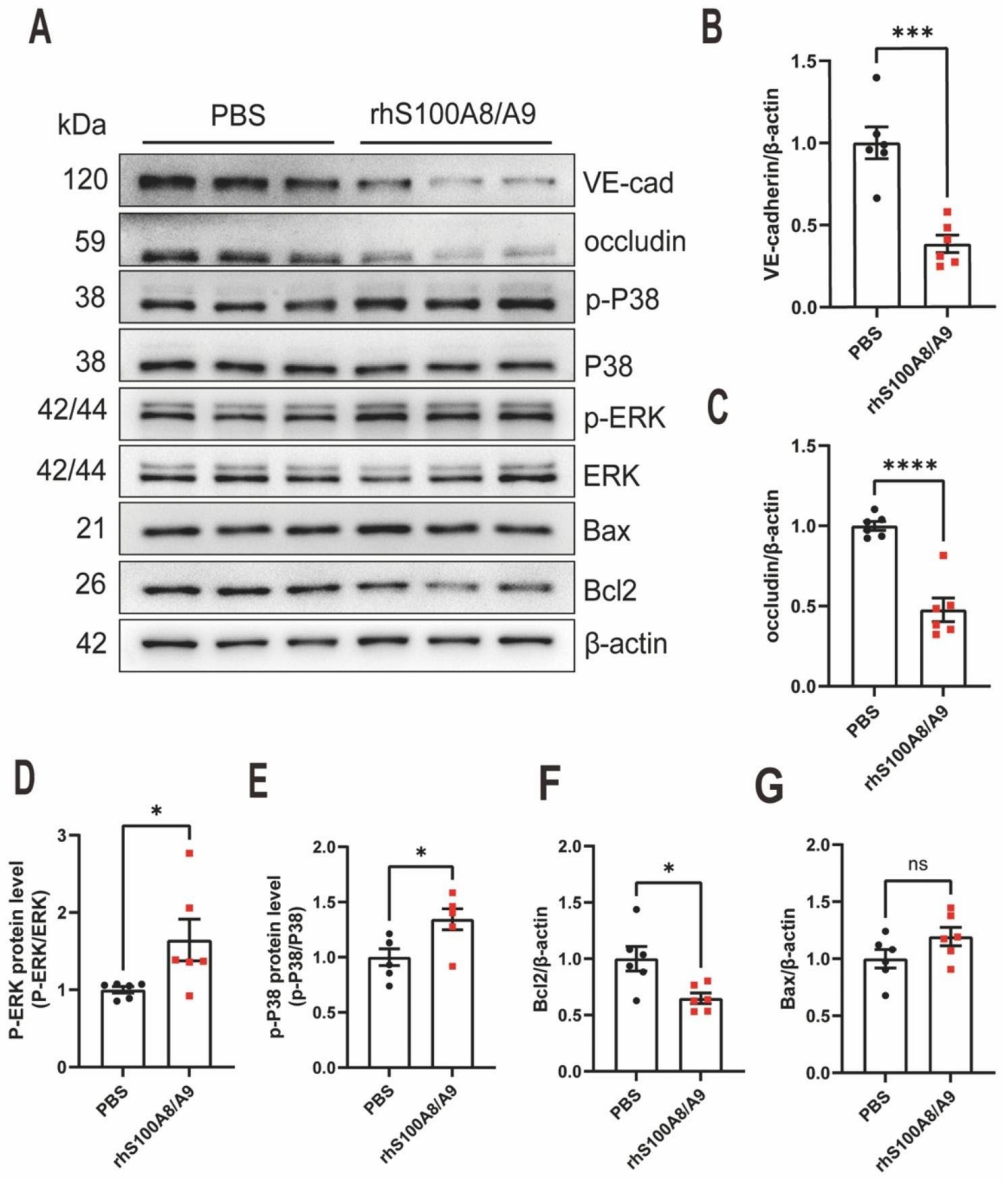
S100A8/A9 promotes endothelial barrier dysfunction in HUVECs. **(A)** Western blot analysis of occludin, VE-cadherin, p-ERK, p-p38, Bax, and Bcl-2 expressions in HUVECs 2 h after rhS100A8/A9 administration (n = 6). The statistical results are presented in **(B-F)**. S100, S100 calcium-binding protein; HUVECs, human umbilical vein endothelial cells; VE, vascular endothelial; p-ERK, phosphorylated extracellular signal-regulated kinase; p-p38, phosphorylated-p38; B-cell leukaemia/lymphoma 2 protein (Bcl-2)-associated X protein; rhS100A8/A9, recombinant S100A8/A9. The data are presented as the mean ± standard error of the mean. *P < 0.05; ***P <0.001; ****P < 0.0001

Subsequently, the role of S100A8/A9 in endothelial barrier dysfunction was assessed using HUVECs. HUVECs were treated with recombinant S100A8/A9 (rhS100A8/A9) and the occludin and VE-cadherin protein levels were examined to achieve this. Results showed that rhS100A8/A9 administration led to a significant reduction in occludin and VE-cadherin expression levels (n = 6) (Fig. 5A-C). Furthermore, it was observed that rhS100A8/A9 administration significantly increased p-ERK and p-p38 levels compared with the untreated controls (n = 6) (Fig. 5A, D-E). Additionally, rhS100A8/A9 treatment significantly decreased the anti-apoptic protein Bcl2 expression however, has no marked influence on Bax expression (n = 6) (Fig. 5A, F). These findings suggest that S100A8/A9-induced activation of the p38/ERK signalling pathway and apoptosis contribute to endothelial cell barrier dysfuntion in HUVECs.

### S100A9 is a candidate biomarker for sepsis diagnosis

**Fig. 6:**
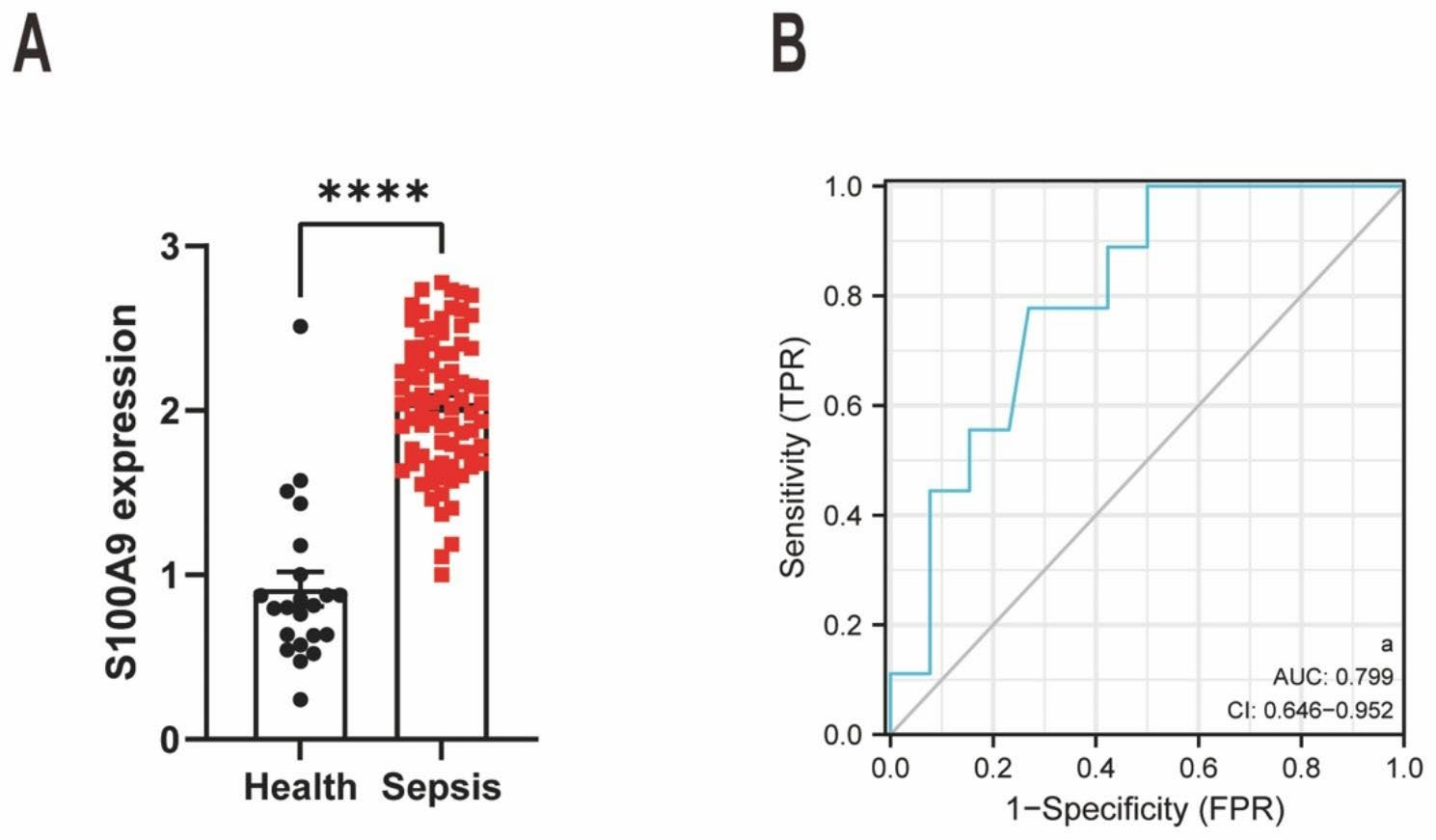
S100A9 is a candidate biomarker for sepsis diagnosis. **(A)** The plasma S100A9 levels in individuals with sepsis (n = 81) compared with healthy controls (n = 22). **(B)** The ROC curve for the diagnostic tests. S100, S100 calcium-binding protein; ROC, receiver operating characteristic. The data are presented as the mean ± standard error of the mean. ****P < 0.0001

A bioinformatics analysis was performed with the help of public databases to determine if the S100A9 plasma levels were elevated in the circulation of patients with sepsis and its clinical value. The findings revealed a significant increase in S100A9 plasma levels in individuals with sepsis (n = 81) compared with healthy controls (n = 22) (Fig. 6A). Furthermore, a receiver operating characteristic curve was constructed to evaluate the sensitivity of S100A9 plasma levels for diagnosing sepsis. The results indicated an AUC of 0.799, suggesting that S100A9 could serve as a diagnostic marker for sepsis (Fig. 6B). These findings suggest that S100A9 could serve as a candidate biomarker for sepsis diagnosis.

## Discussion

The present study revealed a previously unidentified crucial function of S100A8/A9 in inducing pulmonary microvascular hyperpermeability and acute lung injury during sepsis. The study demonstrated that S100A8/A9 expression significantly increased in the lungs of CLP-operated mice. The KO of S100A8/A9 significantly mitigated pulmonary inflammation, vascular leakage, and acute lung injury, resulting in improved survival outcomes in septic mice. These beneficial effects were, at least partially, attributed to the inhibition of p38/STAT3/ERK signalling activation and apoptosis. Furthermore, our findings revealed that S100A9 remained elevated in the circulation of individuals, revealing a strong connection with sepsis diagnosis. These findings provide novel insights into the roles and mechanisms by which S100A8/A9 contribute to pulmonary vascular leakage and acute lung injury and identify S100A8/A9 as a candidate biomarker for sepsis diagnosis.

S100A8 and S100A9 tend to form the S100A8/A9 heterodimer, which has been established as a key player in the development of inflammation [10]. In recent years, growing attention has been focused on the extracellular role of S100A8/A9 as a pro-inflammatory mediator in infectious and non-infectious inflammatory diseases. These include sepsis-induced acute liver injury [33], sepsis-induced cardiomyopathy [20], myocardial infraction (MI) [34], arthritis [35], and psoriasis [36]. These findings align with the pro-inflammatory function attributed to S100A8/A9 in the present study. However, it is important to note that there are reports of anti-inflammatory properties of S100A8/A9. These studies have shown that exogenous S100A8/A9 significantly neutralised the activity of pro-inflammatory cytokines and reduced lipopolysaccharide (LPS)-induced liver [37] and lung injuries [38]. A recent study by Dai et al. [39] revealed that S100A8/A9 might serve as an anti-inflammatory mediator by translocating from the cytosol to the nucleus in Gr1^+^CD11b^+^ myeloid-derived suppressor cells during late sepsis. However, when present in the plasma or secreted by phagocytes, S100A8/A9 might amplify inflammatory processes. Additionally, S100A8/A9 inhibits norepinephrine-induced cardiomyocyte hypertrophy [40]. Short-term blockade of S100A9 could inhibit inflammation and improve cardiac function in mice models of MI [41]. However, long-term blockade of S100A9 could affect cardiac recovery and counteract the beneficial effects of short-term therapy [42]. These findings highlight the complex function of S100A8/A9 in disease, suggesting that it might play a dual role in regulating inflammation homeostasis. S100A8/A9 proteins account for approximately 45% of the cytoplasmic proteins in neutrophils and are actively released during the initial inflammatory phase characterised by predominant neutrophil infiltration [12]. S100A8/A9 might recruit and amplify neutrophil activation during inflammation. S100A8/A9 can regulate neutrophil extracellular trap (NET) formation during sepsis [43]. The NETs can induce the release of S100A8/A9 from neutrophils [44]. This suggests a potential feedback loop where infection-induced neutrophil activation serves as the source of S100A8/A9 secretion in the early stages of sepsis, subsequently activating more neutrophils and increasing the secretion of S100A8/A9, thus perpetuating a vicious cycle. In contrast, analysis of bulk and single-cell sequencing data revealed that the S100A8/A9 gene was highly expressed in monocytes rather than neutrophils in lung tissues of CLP-operated mice [45]. This indicates that monocytes might be the primary subgroup promoting inflammatory responses in septic acute lung injury. Consistent with these findings, our study demonstrated a robust up-regulation of S100A8/A9 expression in the lungs of CLP-operated mice and the circulation of individuals with sepsis. However, the precise mechanism by which sepsis induces S100A8/A9 expression in the lungs remains to be elucidated and requires further investigation in future studies.

Previous studies, including ours, have reported that targeting S100A9 function attenuated sepsis-induced lung damage by inhibiting the inflammatory response [13, 46]. Building upon these findings, the sepsis model of CLP in wildtype and S100A9 KO mice were used for subsequent experimentation, which closely simulated several clinical manifestations of human sepsis [25]. Herein, S100A9 KO effectively ameliorated the clinical symptoms of CLP-operated mice (Fig. 1E, G). Furthermore, the current study primarily focused on evaluating the effect of S100A9 KO on sepsis-induced pulmonary vascular leakage and found that S100A9 KO resulted in decreased pulmonary vascular extravasation of EB. These findings demonstrated the crucial role of S100A8/A9 in disrupting the pulmonary vascular barrier during sepsis (Fig. 2F-G), which, to the best of our knowledge, represents the first study of its kind. However, it needs to point out that the increase of S100A9 levels and vascular leakage may be a mutual causal relationship. In the early stage of sepsis, endotoxin activates leukocyte, which synthesize and secrete S100A8/A9. S100A8/A9 acts on endothelial cells and destroys the vascular endothelial barrier. Vascular leakage might further promote the aggregation of leukocyte in lung tissue, thus leading to more S100A8/A9 in lung tissue. Therefore, blocking the increase of S100A8/A9 may break this vicious cycle and provide a new molecular target for improving vascular hyperpermeability in sepsis.

Notably, p-STAT3 has been implicated in systemic inflammation, acute lung injury, and vascular leakage in sepsis [47–50]. Pharmacological inhibition of p-STAT3 attenuates inflammation, acute lung injury, and endothelial hyperpermeability by inhibiting STAT3 and ERK phosphorylation in the lungs of CLP-operated mice [48]. It has been reported that p38/ERK signalling activation mediates vascular permeability. In the presnet study, it was observed that S100A9 KO inhibited the activities of p38/ERK in the lungs of CLP-operated mice, and rhS100A8/A9 treatment significantly increased p-p38 and p-ERK levels in HUVECs. These findings align with the study conducted by Wang et al. [28], which demonstrated that S100A8/A9 triggers the activation of the MAPK pathway, resulting in cytoskeletal disorganisation and increased HUVECs permeability. This suggests that there is a crosstalk between the STAT3 and p38/ERK signalling pathways in sepsis, and this interaction is associated with acute lung injury and vascular permeability [48]. However, the precise nature of the crosstalk between S100A8/A9 and these signalling pathways in sepsis is yet to be elucidated. The present study is the first to establish that STAT3 signalling is regulated by S100A8/A9 in the lungs of CLP-operated mice, suggestive of a new molecular mechanism underlying sepsis.

This study has several limitations. First, this study solely focused on S100A9 KO mice for investigating the underlying mechanisms, which could exclude potential non-target effects of chemical inhibitors. Therefore, it is crucial to assess the clinical significance of the current findings by further examining the therapeutic potential of drug inhibitors. Second, the precise mechanisms involved in S100A8/A9 up-regulation in the lungs during sepsis and its crosstalk with the STAT3 and P38/ERK signalling pathways warrant further investigation.

## Conclusions

In summary, the present study demonstrated a previously unknown role of S100A8/A9 in promoting pulmonary vascular permeability during sepsis. This effect is mediated, at least partially, through the activation of the P38/STAT3/ERK signalling pathways. Additionally, our findings suggest that S100A8/A9 could serve as a biomarker for sepsis diagnosis.

### Electronic supplementary material

Below is the link to the electronic supplementary material.

Supplementary Material 1: *Title of data: Genotyping of S100A9 null mice. *Description of data: Homozygotes S100A9 null mice had one band with 339 bp, wild-type mice had one band with 557 bp.

## Data Availability

All data, analytical methods, and study materials are available from the corresponding author on request.
