# Supplementary figures and images for: Deficiency of S100A8/A9 attenuates pulmonary microvascular leakage in septic mice
Source: Respir Res. 2023 Nov 17;24:288. doi: 10.1186/s12931-023-02594-0 (PMC10655323; doi:10.1186/s12931-023-02594-0)

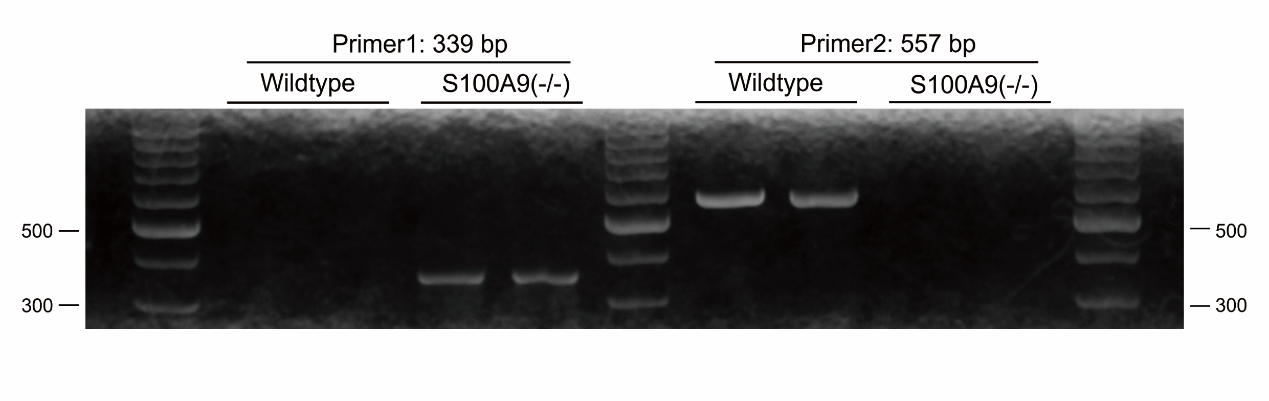
 Fig. S1. Homozygotes S100A9 null mice had one band with 339 bp, wild-type mice had one band with 557 bp.

Supplement: Supplementary file 1 — Supplementary Material 1: *Title of data: Genotyping of S100A9 null mice. *Description of data: Homozygotes S100A9 null mice had one band with 339 bp, wild-type mice had one band with 557 bp. [file 12931_2023_2594_MOESM1_ESM.docx]
